# Body Size Adaptation Alters Perception of Test Stimuli, Not Internal Body Image

**DOI:** 10.3389/fpsyg.2019.02598

**Published:** 2019-11-21

**Authors:** Klaudia B. Ambroziak, Elena Azañón, Matthew R. Longo

**Affiliations:** ^1^Department of Psychological Sciences, Birkbeck, University of London, London, United Kingdom; ^2^School of Advanced Study, The Warburg Institute, University of London, London, United Kingdom; ^3^Department of Experimental Psychology, Institute of Psychology II, Otto-von-Guericke University, Magdeburg, Germany; ^4^Center for Behavioral Brain Sciences, Magdeburg, Germany; ^5^Department of Behavioral Neurology, Leibniz Institute for Neurobiology, Magdeburg, Germany

**Keywords:** body representations, visual perception, sensory adaptation, self, aftereffects

## Abstract

Recent studies have reported that adaptation to extreme body types produces aftereffects on judgments of body normality and attractiveness, and also judgments of the size and shape of the viewer’s own body. This latter effect suggests that adaptation could constitute an experimental model of media influences on body image. Alternatively, adaptation could affect perception of test stimuli, which should produce the same aftereffects for judgments about participant’s own body or someone else’s body. Here, we investigated whether adaptation similarly affects judgments about one’s body and other bodies. We were interested in participants’ own body image judgments, i.e., we wanted to measure the mental representations to which the test stimuli were compared and not the perception of test stimuli *per se*. Participants were adapted to pictures of thin or fat bodies and then rated whether bodies were fatter or thinner than either: their own body, an average body (Experiment 1), or the body of another person (Experiments 2 and 3). By keeping the visual stimuli constant but changing the task/type of judgment, i.e., the internal criterion participants are asked to judge the bodies against, we investigated how adaptation affects different stored representations of bodies, specifically own body image vs. representations of others. After adaptation, a classic aftereffect was found, with judgments biased away from the adapting stimulus. Critically, aftereffects were nearly identical for judgments of one’s own body and for other people’s bodies. These results suggest that adaptation affects body representations in a generic way and may not be specific to the own body image.

## Introduction

In our daily lives, we constantly experience our own bodies directly through touch and proprioception, but most of our visual experience with bodies comes from seeing other people. For most of us, other people’s bodies are a ubiquitous part of our “visual diet” (Boothroyd et al., 2012). Moreover, through the media, we are bombarded with images of other, often idealized, bodies. Previous research showed that exposure to thin, idealized images changes attitudes toward one’s own body, increases body dissatisfaction, and negatively affects mood (Groesz et al., 2002; Tiggemann and McGill, 2004). Clearly, media can shape our beliefs and attitudes, but can exposure to certain body types change the way we actually *perceive* our own bodies? Recently, several studies suggested that this might be the case and proposed visual adaptation, i.e., a shift in perceptual judgment after prolonged exposure to a certain stimulus, as one of the mechanisms that may be involved in this process (Hummel et al., 2012b; Brooks et al., 2016; Bould et al., 2018; Stephen et al., 2018). According to this emerging theory, exposure to idealized, often extremely thin bodies (e.g., “size zero” models) causes our own body to be perceived as fatter than it really is. Alternatively, adaptation to extreme body types might affect the way people visually perceive test stimuli, without affecting the viewer’s own body image at all. Here, we further investigated whether short-term effects of adaptation could constitute an experimental model for the long-term effects of media influences on body image, by asking whether perceptual changes induced by body size adaptation show specificity to one’s own body or, on the contrary, are similar across visual representations of bodies in general. We were interested in body image judgments, i.e., we wanted to measure the mental representations to which the test stimuli were compared.

Body representations arise from many different sources: interoceptive signals from internal organs; sensory input from the external world coming from modalities such as touch and vision; and also from abstract knowledge, beliefs, and attitudes (see Longo et al., 2010 for a review). The body image is a complex concept that involves both sensory and cognitive components. While many body representations are thought to be largely based on somatosensory signals about touch, proprioception, pain, etc. (Longo, 2015), the body image is thought to be predominantly visual. Indeed, in his classic definition, Schilder (1935/1950) used overtly visual language in describing the body image as “the picture of our own body which we form in our mind, that is to say the way in which the body appears to ourselves” (p. 11). Although, in the literature, body image is often used in reference to attitudes and feeling about one’s body, here we wanted to specifically focus on its visual component, i.e., the way the body appears to us in the mind’s eye. As a predominantly visual representation, is the body image then shaped by visual exposure to other bodies?

Visual adaptation produces aftereffects which bias perception in the direction opposite to the adapting stimulus. For example, in the classic waterfall illusion, after a short exposure to the flowing water of a waterfall, when the observer looks at a static image, e.g., the space beside the waterfall, she will perceive it as moving upward (Barlow and Hill, 1963; Anstis et al., 1998). Visual adaptation aftereffects are widely studied and well established for basic features, such as motion (Wohlgemuth, 1911), orientation (Gibson and Radner, 1937), and color (McCollough, 1965), which are thought to be processed mainly at lower levels of visual hierarchy. However, research on adaptation to complex stimuli such as faces and bodies suggests that adaptation is not limited to basic features and may operate at higher levels of processing (Rhodes et al., 2003; Webster et al., 2004; Fox and Barton, 2007; Pond et al., 2013; Brooks et al., 2018). Research on face adaptation shows that adaptation to faces with consistent distortions, i.e., compressing or expanding the center of a face, causes normal faces to appear distorted in the opposite direction (Rhodes et al., 2003). In a similar way, gender-ambiguous faces are judged as more masculine after prolonged viewing of female faces, whereas adaptation to male faces induces the opposite effect (Webster et al., 2004; Pond et al., 2013). Facial emotional expressions also produce similar aftereffects: after exposure to happy faces, observers tend to perceive subsequent neutral expressions as sad (Fox and Barton, 2007).

Research on body adaptation has shown that brief exposure to unfamiliar thin bodies significantly alters people’s perception of body attractiveness, normality, and ideals, in the direction of the thin adaptor (Winkler and Rhodes, 2005; Glauert et al., 2009). Moreover, adaptation to participants’ own bodies, depicting them as either thinner or fatter, can also alter the way participants judge images of their own bodies. After adaptation to the thin version of their own body, participants rated a thinner than actual image to be the most accurate depiction of their own body and vice versa for the fat adaptor (Hummel et al., 2012a,b, 2013). Interestingly, the effect of body adaptation transfers across identities, with comparable effects for unfamiliar and own body adaptors (Hummel et al., 2012b; Brooks et al., 2016). Moreover, it is specific to bodies, and does not transfer between bodies and narrow/wide rectangles Hummel et al. (2012a). Taken together, these findings show that exposure to thin images not only affects perceived norms and ideals (Winkler and Rhodes, 2005; Glauert et al., 2009) but can also change how participants judge images of their own body – causing the actual image of the participant’s body to appear fatter (Hummel et al., 2012b; Brooks et al., 2016). These results suggest that visual adaptation may serve as an experimental model of the effect that exposure to thin bodies presented in media has on body image. Following this line of reasoning, Challinor et al. (2017) proposed that including visual adaptation as a part of treatment may have therapeutic effect on patients with body image distortions in conditions such as anorexia nervosa.

Here, we investigated whether adaptation affects visual body image in a self-specific way. There is clear evidence that body size adaptation changes perception of bodies as indicated by the shift in perceived norms as the result of aftereffects. The question nevertheless remains how similar the magnitude of the aftereffects is for one’s own body (i.e., the body image) and for bodies in general. Specifically, it is not known whether exposure to bodies affects judgments about our own body in a self-specific way, or the effect is generic to all bodies. For visual adaptation to constitute an experimental model of media effect on body image distortions, some overlap in the way adaptation affects one’s own body and bodies of others is required to allow the transfer of aftereffects from media images to body image as it has been argued previously (Brooks et al., 2016). However, the effect that adaptation has on one’s own body should also differ from the general effect of adaptation on all images of bodies. If adaptation affects all bodies equally, the relative difference between one’s own body and other bodies should not change. In other words, both our own bodies and bodies presented in media should be affected by adaptation. In consequence, our own body should not appear to us as fatter in the whole continuum of bodies, i.e., if we were to compare our body to other bodies. Interestingly, there are many clinical conditions in which people have distorted image of their own body, but not other people’s (whereas the opposite is rare). Studies have reached divergent conclusions about whether patients with eating disorders selectively overestimate the size of their own bodies (e.g., Mizes, 1992; Øverås et al., 2014) or overestimate bodies in general (e.g., Tovée et al., 2000; Horndasch et al., 2015). In a study by Guardia et al. (2012), patients showed biased judgments about their own actions but could accurately judge the affordances of others. These results suggest that anorexia nervosa patients do not have distorted representations of other people’s bodies.

To investigate whether visual exposure to extreme body types affects the perception of our own body and of other bodies in similar or different ways, we designed three experiments in which female participants judged the same test images but compared them to different internal representations. After adaptation to pictures of either extremely thin or extremely fat bodies, participants were asked to rate whether subsequently presented bodies were fatter or thinner than either: their own body (all three experiments), an average body (Experiment 1), or a body of a specific other person (Experiments 2 and 3). By keeping the visual stimuli constant but changing the internal criterion (or anchor point) that participants are asked to judge the bodies against, we investigated how adaptation affects different stored representations of bodies, specifically, and not the perception of the test stimuli *per se*. Note that in a body image judgment, the reference (a photograph, a silhouette, or even a piece of string) is usually compared to one’s mental representation of one’s own body, regardless of the nature of the reference itself (e.g., Slade and Russell, 1973; Garner et al., 1976; Thompson et al., 1986; Schneider et al., 2009). Interestingly, a previous study by de la Rosa et al. (2014) reported modulation of action adaptation aftereffects across two conditions that had identical adapting and test stimuli but differed with respect to the information that was provided prior to the adaptation, i.e., the social context, suggesting that the context of the task can affect adaptation aftereffects.

## Experiment 1

In Experiment 1, we investigated the effect of visual adaptation to thin bodies on judgments about one’s own body and about an average body. If adaptation has a specific effect on body image, the magnitude of adaptation aftereffects should differ depending on whether participants are comparing the test image to their own body or somebody else’s. If, in contrast, adaptation affects body image judgments by altering perception of the test stimuli, identical aftereffects should be found in both cases.

### Methods

#### Participants

Due to the nature of our stimuli (depicting female bodies), we restricted our sample to female participants. A different group of participants was selected in each study. Twenty participants (mean age: 28.1, SD: 11.6, range: 18–65; mean body mass index, BMI: 21.9, SD: 3.1, range 17.9–29.1) took part in Experiment 1. All participants had normal or corrected to normal vision. All participants gave informed consent and were paid for their participation. The procedures were approved by the ethics board of the Department of Psychological Sciences, Birkbeck, University of London.

Previous studies showed that adaptation to bodies produces strong and robust effects. For example, Hummel et al. (2012b) using similar type of stimuli and similar type of judgments in two experiments obtained Cohen’s *d*_z_’s of 2.87 and 1.04. We conducted a power analysis using G*Power 3.1 taking the smaller of these two effect sizes, an alpha value of 0.05 and power of 0.95, which indicated that 12 participants were required. In addition, piloting data using our paradigm showed adaptation effects in virtually every participant. Thus, we believe that our sample size of 20 participants makes this experiment well powered to address this issue.

#### Stimuli

**Figure 1:**
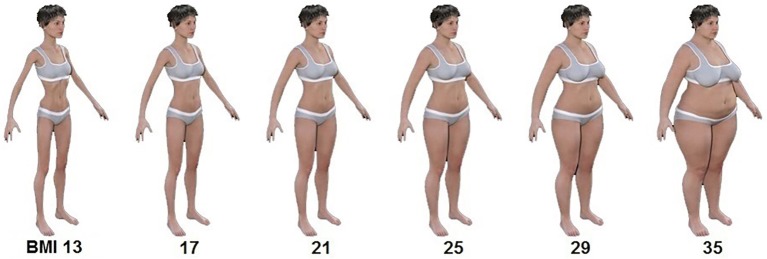
Stimuli used in the experiments. A continuum of 89 body shapes was created, ranging from extremely thin (i.e., BMI = 13) to obese (i.e., BMI = 35). 3D modeling software was used to model these changes in a biologically realistic way. The numbers indicate the estimated body mass index (BMI) of the avatars.

We used a set of 89 images of female bodies rendered from 3D avatars generated in DAZ Studio 4.8 (DAZ Productions, http://www.daz3d.com/). Avatars’ BMI ranged from 13 (emaciated) to 35 (obese) with an increment of 0.25 BMI units between each stimulus (with a total of 89 images; see Figure 1 for examples). To approximate the avatars’ BMI, we used Cornelissen et al.’s (2009) formula to calculate the waist-to-hip ratio (WHR) for white UK women of reproductive age: WHR = (2.057 × BMI + 29.67)/(1.842 × BMI + 56.004), which is based on data from Health Survey for England (2003). Following this formula, waist and hip circumferences were estimated for each required BMI from the range of 13–35. Avatars’ waist and hip circumferences were adjusted using the Universal Sizing Apparatus tool (Rocketship Technologies Inc., http://rocketship3d.com/). The height of the avatars was kept constant at 170 cm. The avatars were rotated approximately 45° around the vertical axis (in the transverse plane) to obtain a viewing angle that would provide more information about the body dimensions compared to the straight-facing view. A recent study by Cornelissen et al. (2018) confirmed that this three-quarter view results in the most accurate estimations of body size. Finally, 2D images were rendered from the avatars, as shown in Figure 1.

#### Procedures

Each experiment consisted of the *Baseline* and the *Adaptation* phase conducted on the same day with a short break (1–2 min). In Experiments 1 and 2, a very thin body (BMI = 13) was used as an adapting stimulus. In Experiment 3, the procedure was repeated twice, using both a very thin (BMI = 13) and a very fat adaptor (BMI = 35). In all experiments, participants sat approximately 50 cm from the screen with head movements unrestricted. Images were presented in the center of a 24-inch screen (resolution: 1,600 × 1,200 pixels; refresh rate: 75 Hz), on a black background. The height of each image was 18 cm (20° visual angle). Stimuli were presented using Psychtoolbox (Brainard, 1997), running on MATLAB (Mathworks, Natick, MA, USA). The PSEs were calculated using a Bayesian adaptive algorithm QUEST (Watson and Pelli, 1983). Statistical analyses were performed in JASP (JASP Team, 2017).

In the Baseline phase, each trial began with a blank screen (1,000 ms), followed by a fixation cross (1,000 ms) that turned from black to red to indicate the beginning of a new trial. Then, a question appeared on the screen: *Is this body fatter than your own?* in the *Self* condition, or *Is this body fatter than average?* in the *Average* condition. Before the start of the task, we explained to participants that “average” means the most common/typical body for their age and gender (according to their best guess). The question was followed by a 1,000-ms test body selected by QUEST, from a set of possible stimuli based on their history of responses on previous trials. A blank screen remained visible until the response was made using labeled keyboard keys (“yes”/“no”). After the response, a black cross appeared on the screen for 1,000 ms indicating the end of the trial. Each part was divided in four blocks of 40 trials (two blocks per condition, ABBA order counterbalanced across participants). At the end of each block, the point of subjective equality (PSE, i.e., the stimulus for which the participant was equally likely to judge it as fatter or thinner) was calculated using QUEST. The PSEs from the two blocks were then averaged to obtain a single estimate for each condition, separately for the Baseline and the Adaptation phase.

The Adaptation phase started with an initial 2-min exposure to the thin adaptor. The adaptor flickered every 4 s (disappearing for 500 ms and appearing again) to maintain attention. After that, participants performed the same task as in the Baseline phase. Each trial in the Adaptation phase was identical to the Baseline with the addition of a thin body exposure. A very thin adaptor (BMI 13) was presented for 8 s to “top-up” the adaptation, followed by one second of blank screen, just before the presentation of the corresponding question (i.e., *is this body fatter than your own/average*) and the test stimulus. Again, the PSE was calculated after every block, resulting in two PSEs per condition.

### Results

**Figure 2:**
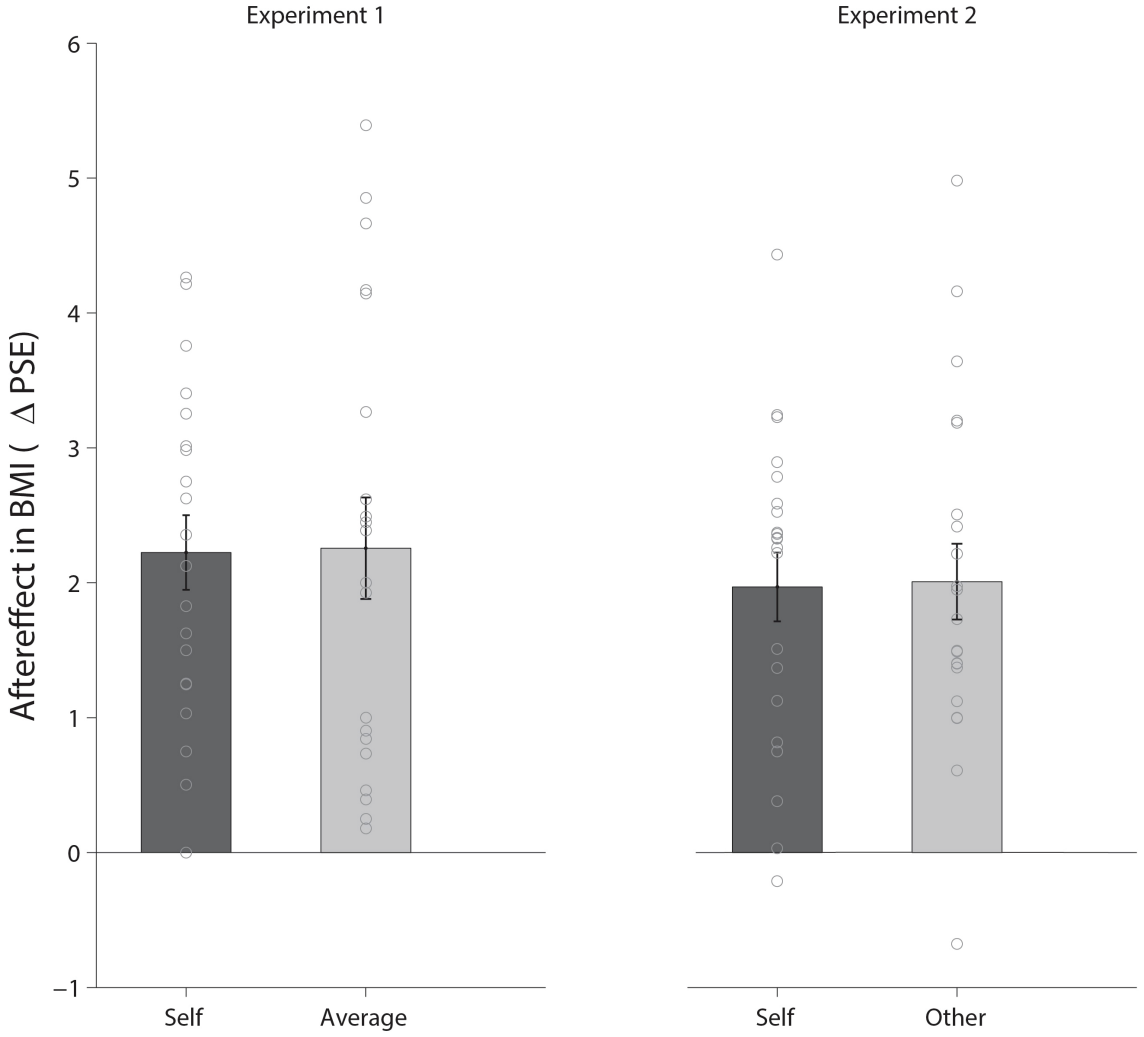
Results of Experiments 1 (left panel) and 2 (right panel). (Left panel) The effect of adaptation for the *Self* and *Average* conditions shown as the pre-post adaptation shift in PSE. The dots indicate individual subjects, and the error bars represent standard errors. Clear adaptation aftereffects were apparent for both body image judgments in the *Self* condition and judgments of typicality in the *Average* condition. The magnitude of the aftereffects was very similar in the two conditions. (Right panel) The effect of adaptation for the *Self* and *Other* conditions presented in the same way as in Experiment 1.

In each experimental session, two 50% thresholds (PSEs) per condition were calculated using QUEST to estimate the BMI at which participants were equally likely to respond thinner or fatter. These two PSEs were then averaged, resulting in four PSEs, one for each condition (*Self*, *Average*) and adaptation phase (*Baseline* and *Adaptation*). Our main effect of interest was *the pre-post adaptation shift* of the PSEs in Self and Other condition. The results are shown in Figure 2 (left panel). Clear adaptation aftereffects were apparent in both conditions. In the Self condition, the mean perceived BMI decreased from 28.1 (SD = 2.3) at pre-test to 25.9 (SD = 2.8) after adaptation, Similarly, in the Average condition, mean judgments decreased from 28.5 (SD = 1.9) at pre-test to 26.3 (SD = 2.2) after adaptation. This clear decrease in perceived BMI from *Baseline* to *Adaptation* was significant, both in the *Self* condition (mean change: 2.22, SD: 1.24), *t*(19) = 8.05, *p* < 0.001, *d*_z_ = 1.80, and in the *Average* condition (mean change: 2.26, SD: 1.68), *t*(19) = 6.00, *p* < 0.001, *d*_z_ = 1.34.

To investigate the effects of the two judgment types, we conducted a 2 × 2 repeated measures analysis of variance (ANOVA) on the PSEs with factors *condition* (*Self* / *Average*) and *adaptation* (*Baseline* / *Adaptation*). We found a main effect of adaptation, *F*(1, 19) = 50.78, *p* < 0.001, $\eta_{p}^{2}$ = 0.75. That is, after adaptation to a thin adaptor, participants perceived as fatter, images that before adaptation were considered as thinner (see Figure 2). There was no effect of condition, *F*(1, 19) = 0.74, *p* = 0.4, $\eta_{p}^{2}$ = 0.04, suggesting that on average participants did not judge themselves as fatter or thinner than a typical woman. Critically, there was no interaction, *F*(1, 19) = 0.026, *p* = 0.874, $\eta_{p}^{2}$ = 0.00, suggesting that adaptation affected participants’ perception of themselves in the same way it affected perception of typicality. Moreover, the magnitude of the aftereffects in both conditions was correlated across participants, *r* = 0.85, *p* < 0.001.

There was a correlation between participants’ own BMI and the baseline PSEs in the Self condition: *r* = 0.71, *p* < 0.001, but not in the average condition: *r* = 0.16, *p* = 0.500. Similarly, after adaptation, there was a correlation between participants’ own BMI and PSEs in the Self condition: *r* = 0.69, *p* < 0.001, but not in the Other condition: *r* = 0.35, *p* = 0.128. The clear correlation between actual and perceived BMI is important to note given that other tasks purporting to measure body image (such as the moving caliper method) have been criticized on the basis that no such correlations were apparent (e.g., Ben-Tovim and Crisp, 1984; Ben-Tovim et al., 1990).

To further compare the magnitude of the aftereffects in two conditions, we conducted a Bayesian paired *t*-test (Rouder et al., 2009), comparing the PSE change (Baseline/Adaptation) between conditions, which provided support for the null hypothesis, BF_(0, 0.7)_ = 0.24.

The absence of an overall effect of *Self* vs. *Average* condition may suggest that participants genuinely considered themselves as being about the average size, despite the fact that the average BMI of our participants (21.9) was lower than the UK average for females in the same age range, which was reported to be 25.9 (Health Survey for England, 2016). Alternatively, the results may suggest that participants did not perform different tasks in the two conditions and possibly used themselves as a reference in both. Experiment 2 aimed to address these issues.

## Experiment 2

The results of Experiment 1 revealed no differences between the *Self* and *Average* conditions. This therefore did not allow us to conclude that participants actually performed two different tasks in those two conditions. In Experiment 2, instead of the *Average* condition we designed a condition (*Other*), in which participants had to compare test stimuli with a specific person, namely the experimenter (KBA) with whom participants interacted prior to performing the task. We reasoned that the use of a specific person rather than an abstract “average” person would make the task easier and clearer to the participants.

### Methods

#### Participants

Twenty-one participants (mean age: 29.9, SD: 10, range: 19–58; mean BMI: 23.7, SD: 4.8, range 17.0–35.0) took part in Experiment 2. All participants had normal or corrected to normal vision. All participants gave informed consent and were paid for their participation. The procedures were approved by the ethics board of the Department of Psychological Sciences, Birkbeck, University of London.

#### Stimuli and Procedures

Stimuli were identical to Experiment 1. Procedures were similar to Experiment 1. This time, however, participants were asked to compare the images either with themselves, answering the question: *Is this body thinner or fatter than your own?*, or with the experimenter (KBA), answering the question: *Is this body thinner or fatter than Klaudia?* (*Other* condition). Before the start of the experiment, participants had approximately 5 min of visual experience of the experimenter while she introduced the task. The experimenter was wearing close-fitting clothes and after explaining the task, she stood in front of the participant and asked them to memorize her body size and shape for about 10 s. During the task, participants were not looking at the experimenter. As in Experiment 1, the experiment consisted of two parts: *Baseline* and *Adaptation*. Each part was divided into four blocks of 30 trials (two blocks per *Self*/*Other* condition, in ABAB order, counterbalanced across participants). To shorten the length of the experiment, the duration of the adapting stimuli was reduced to 6 s, and the duration of the initial blank screen and the fixation cross before and after the response was reduced to 500 ms each.

### Results and Discussion

Results from Experiment 2 are shown in Figure 2 (right panel). In the *Self* condition, mean perceived BMI decreased from 25.7 (SD = 3.4) at pre-test to 23.7 (SD = 3.8) after adaptation. Similarly, in the *Other* condition, PSEs decreased from 24.3 (SD = 1.7) at pre-test to 22.3 (SD = 1.9) after adaptation. Again, clear adaptation aftereffects were significant in both the *Self* condition (mean change: 1.97, SD: 1.17), *t*(20) = 7.71, *p* < 0.001, *d*_z_ = 1.68, and the *Other* condition (mean change: 2.01, SD: 1.29), *t*(20) = 7.15, *p* < 0.001, *d*_z_ = 1.56^1^. A 2 × 2 repeated measures ANOVA with factors condition and adaptation revealed a main effect of adaptation, *F*(1,20) = 81.47, *p* < 0.0001, $\eta_{p}^{2}$ = 0.80, showing that exposure to a thin body affected the perception of test bodies. We also found a significant main effect of condition, *F*(1, 20) = 5.30, *p* = 0.032, $\eta_{p}^{2}$ = 0.21, which reflects the fact that the experimenter was perceived differently (overall as thinner) than the participants perceived themselves. This difference between the two types of judgments is important as it demonstrates that participants were in fact making different judgments in the two conditions. Critically, however, there was no significant interaction, *F*(1, 20) = 0.02, *p* = 0.9, $\eta_{p}^{2}$ = 0.00, again suggesting that adaptation affected participants’ perception of themselves in the same way it affected perception of the experimenter’s body. As in Experiment 1, there was a positive correlation between the magnitude of the aftereffects in the two conditions, though it did not differ significantly from 0, *r* = 0.35, *p* = 0.12.

There was a correlation between participants’ own BMI and the baseline PSEs in the Self condition: *r* = 0.69, *p* < 0.001, but not in the Other condition: *r* = 0.08, *p* = 0.730. After adaptation, there was a correlation between participants’ own BMI in both conditions: *r* = 0.72, *p* < 0.001 in the Self condition and *r* = 0.49, *p* = 0.024 in the Other condition, respectively.

As in Experiment 1, a Bayesian paired *t*-test comparing the magnitude of the change in PSE in the two conditions provided additional support for the null hypothesis: BF_(0, 0.7)_ = 0.23, further suggesting that there is no difference in the magnitude of aftereffects between the *Self* and the *Othe*r condition.

These results showed that adaptation to an extremely thin body affected judgments about self vs. other body similarly and therefore the magnitude of the aftereffects was not influenced by the type of judgment being made. However, previous research suggested that in some cases body adaptation may be affected by the task. Winkler and Rhodes (2005) showed that while adaptation to a thin body had an effect on both perceived normality and attractiveness of test bodies, adaptation to a fat body did not significantly affect perceived attractiveness. In Experiment 3, we tested whether adaptation to fat bodies affected self vs. other body judgments equally.

One possible limitation of Experiment 2 is the fact that the experimenter was used as a reference in the Other condition. Although the pattern of results in this condition resembles typical adaptation aftereffects, it is possible that the responses were affected by some form of participant bias or social desirability bias in which participants change their responses to more socially acceptable. Since judging another person’s weight is a sensitive task and participants knew that the experimenter would eventually see their responses, it is possible that they altered their judgments. Therefore, in Experiment 3, in which we tested the effect of both thin and fat exposure, we used a famous person (Kate Middleton) as a reference in the Other condition.

## Experiment 3

Experiment 3 aimed to test the effect of thin and fat adaptation on judgments about self and other bodies. This time, in the *Other* condition, we used a famous person in the United Kingdom, i.e., Kate Middleton (the Duchess of Cambridge). We chose Kate Middleton as we expected that she would be familiar to a largest group of potential participants. In addition, KM has a BMI of about 18 which is lower than that of an average UK female (and indeed lower than that of 90% of participants in Experiments 1 and 2), which made the difference between the conditions most apparent.

### Methods

#### Participants

Eighteen participants took part in Experiment 3 (mean age: 27.1, SD: 7.3, range: 20–41; mean BMI: 20.2, SD: 2.2, range 17.0–25.4). Two additional participants who signed up for Experiment 3 were tested but their data were never analyzed. One of them was pregnant and the other one had a BMI beyond the range of our stimuli. All participants had normal or corrected to normal vision. All participants gave informed consent and were paid for their participation. The procedures were approved by the ethics board of the Department of Psychological Sciences, Birkbeck, University of London.

#### Stimuli and Procedures

Procedures were similar to those of Experiment 2. However, in the *Other* condition, participants were asked to compare the test images with Kate Middleton, answering the question: *Is this body thinner or fatter than Kate Middleton?* We made sure that all participants were familiar with the appearance of Kate Middleton prior to the experiment. Additionally, at the beginning of the experiment, participants were presented with five full body images of Kate Middleton.

Unlike the first two experiments which involved only a thin adapting stimulus, Experiment 3 included both a thin (BMI 13) and a fat adaptor (BMI 35). The experiment therefore consisted of four parts: Baseline and Adaptation, each repeated twice, once with a thin and once with a fat adaptor. Each of these four parts was further divided in four blocks of 36 trials (two blocks per *Self*/*Other* condition, in ABAB order, counterbalanced across participants). To further reduce the length of the experiment, initial adaptation was shortened to 1 min, the top-up adaptation to 4 s, and the initial blank screen to 250 ms. Participants took a 10-min break between the two adaptation procedures (i.e., thin and fat) to allow the effect of adaptation to wear off. The order of thin/fat adaptation was counterbalanced across participants.

### Results and Discussion

**Figure 3:**
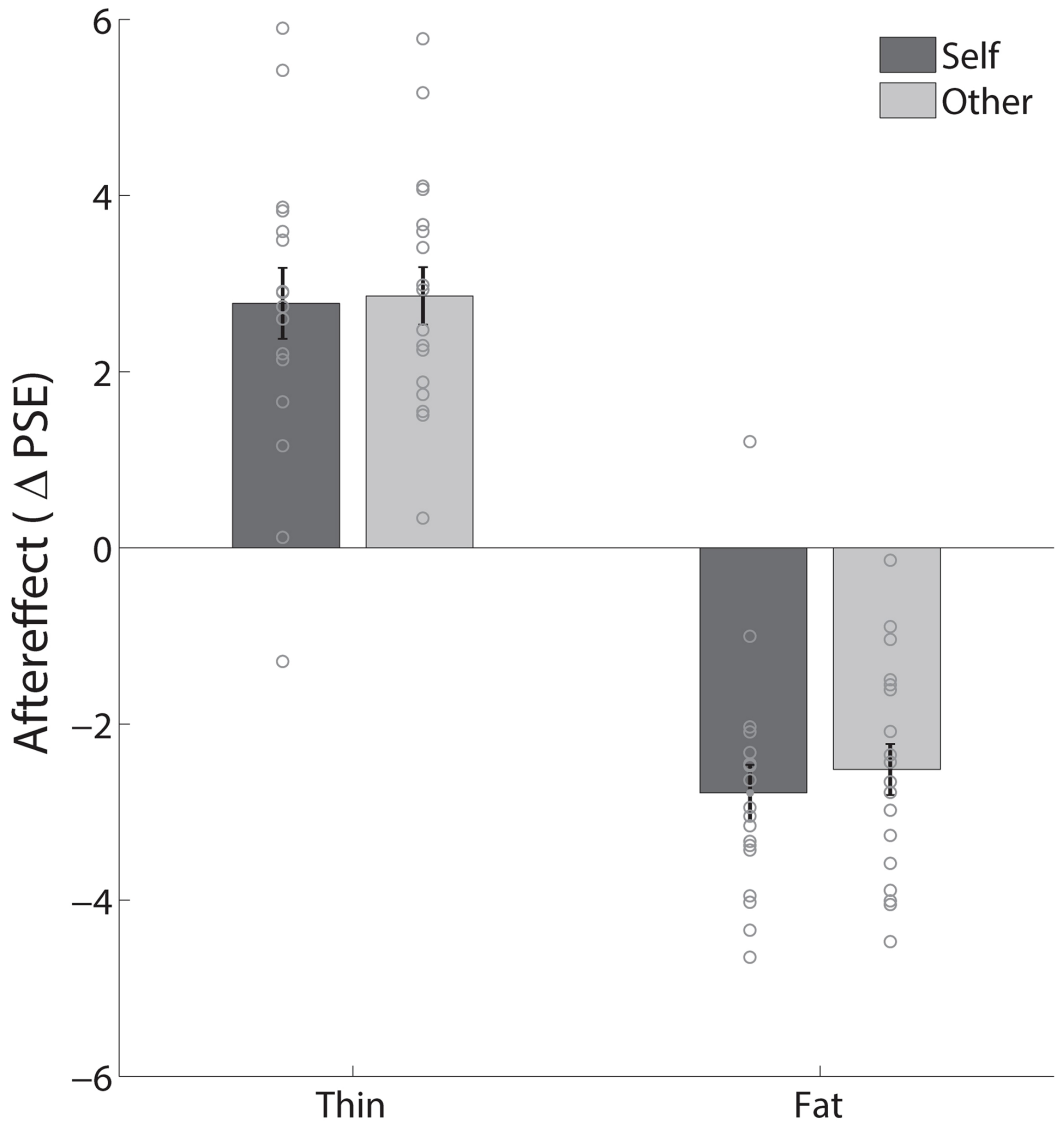
Results of Experiment 3. The effect of thin and fat adaptation for *Self* and *Other* condition. The gray dots indicate individual subjects and the error bars represent standard errors.

The results from Experiment 3 are shown in Figure 3. After adaptation to a thin body, in the *Self* condition, the mean perceived BMI dropped from 23.8 (SD = 2.6) at pre-test to 21.0 (SD = 3.0) after adaptation. In the *Other* (i.e., Kate Middleton) condition, perceived BMI dropped from 21.97 (SD = 1.7) at pre-test to 19.11 (SD = 1.24) after adaptation. As in the first two experiments, clear aftereffects were apparent in both the *Self* condition (mean change: 2.78, SD: 1.71), *t*(17) = 6.89, *p* < 0.001, *d*_z_ = 1.63, and the *Other* (i.e., Kate Middleton) condition (mean change: 2.86, SD: 1.39), *t*(17) = 8.76, *p* < 0.001, *d*_z_ = 2.07. Similar aftereffects were also found after adaptation to a fat body. In the Self condition, the mean perceived BMI increased from 22.6 (SD = 2.3) at pre-test to 25.4 (SD = 2.4) after adaptation. In the Other condition, perceived BMI increased from 21.5 (SD = 1.6) at pre-test to 24.0 (SD = 1.8) after adaptation. There were clear increases in judged BMI in the *Self* condition (mean change: −2.78, SD: 1.34), *t*(17) = −8.76, *p* < 0.001, *d*_z_ = 2.07, and the *Other* condition (mean change: −2.51, SD: 1.23), *t*(17) = −8.67, *p* < 0.001, *d*_z_ = 2.04. Thus, clear aftereffects were found for both thin and fat adapting stimuli, both for judgments of one’s own body and of Kate Middleton’s body.

A 2 × 2 × 2 repeated measures ANOVA with factors *condition* (*Self*/*Other*), *adaptation* (*Baseline*/*Adaptation*), and *adapting body type* (thin/fat) was performed on the PSEs. We found no main effect of adaptation, *F*(1,17) = 0.22, *p* = 0.644, $\eta_{p}^{2}$ = 0.01. There was, however, a main effect of adapting body type, *F*(1,17) = 102.34, *p* < 0.001, $\eta_{p}^{2}$ = 0.86, and an interaction between adaptation and adapting body type, *F*(1,17) = 187.26, *p* < 0.001, $\eta_{p}^{2}$ = 0.92, showing that adaptation to thin vs. fat bodies produced strong effects in opposite directions. We tested this assumption using two-tailed paired *t* tests directly comparing results of adaptation sessions to thin vs. fat: *t*(17) = −11.78, *p* < 0.0001, *d*_z_ = −2.72 for the *Self* condition, and *t*(17) = −12.25, *p* < 0.0001, *d*_z_ = −2.89 for the *Other* condition. We also found a main effect of *Self*/*Other* condition, *F*(1,17) = 11.65, *p* = 0.003, $\eta_{p}^{2}$ = 0.41, clearly indicating that participants in the study perceived their bodies as different from Kate Middleton’s (overall as fatter). The interaction between the *Self*/*Other* condition and the adapting body type (thin/fat) was also significant, *F*(1,17) = 5.76, *p* = 0.028, $\eta_{p}^{2}$ = 0.25. However, again there was no interaction between adaptation and *Self*/*Other* condition, *F*(1,17) = 0.306, *p* = 0.587, $\eta_{p}^{2}$ = 0.018, and no interaction between all three factors, *F*(1,17) = 0.132, *p* = 0.721, $\eta_{p}^{2}$ = 0.08. This indicates that adaptation affects participants’ judgments of themselves in the same way it affected judgments of Kate Middleton’s body. As in Experiments 1 and 2, there was a positive correlation between the magnitude of the aftereffects in the two conditions, though it did not differ significantly from 0: *r* = 0.33, *p* = 0.18 for the thin adaptation and *r* = 0.22, *p* = 0.38 for the fat adaptation.

In the thin adaptation, there was a correlation between participants’ own BMI and the baseline PSEs in the Self condition: *r* = 0.66, *p* = 0.003, but not in the Other condition: *r* = 0.20, *p* = 0.426. Similarly, after adaptation, there was a correlation between participants’ own BMI and PSEs in the Self condition: *r* = 0.77, *p* < 0.001, but not in the Other condition: *r* = 0.25, *p* = 0.308. In fat adaptation, there was a correlation between participants’ own BMI and the baseline PSEs in the Self condition: *r* = 0.72, *p* < 0.001, but not in the Other condition: *r* = 0.03, *p* = 0.906. Similarly, after adaptation there was a correlation between participants’ own BMI and PSEs in the Self condition: *r* = 0.70, *p* = 0.001, but not in the Other condition: *r* = 0.05, *p* = 0.845.

Again, the results of the Bayesian paired *t*-test comparing the magnitude of the aftereffects in two conditions showed that data support the null hypothesis for both, thin adaptor, BF_(0, 0.7)_ = 0.25, and fat adaptor, BF_(0, 0.7)_ = 0.30.

## General Discussion

Body dissatisfaction is a prevalent problem in modern societies (Grogan, 2017). Media are often blamed for creating unrealistic, unhealthy body ideals that can shape our beliefs and attitudes (Derenne and Beresin, 2006). Recently, it has also been suggested that exposure to thin ideals can influence the way we actually perceive our own bodies. Several studies (Hummel et al., 2012b; Brooks et al., 2016) proposed visual adaptation as a model of media influences on one’s own body image. Our results replicate previous results showing that visual adaptation to extreme body types affects body image judgments. Critically, however, virtually identical effects were found for judgments of other people’s bodies. This suggests that adaption does not have specific effects on the participant’s body image. We suggest instead that adaptation may have affected body types of judgment by changing visual perception of the test stimuli. Here, we showed that adaptation affects judgments about one’s own body in a similar way as judgments about other people’s bodies, both when asking about typical bodies (Experiment 1), or about a specific other person’s body (Experiments 2 and 3). Importantly, we found a main effect of *Self*/*Other* condition in Experiments 2 and 3, indicating that people were indeed performing different tasks when comparing the test stimuli with either their own body or bodies of others.

Our findings are consistent with the results of previous studies that reported transfer of body size aftereffects between different identities (Hummel et al., 2012b; Brooks et al., 2016). The authors of these studies suggest that their results reflect perceptual bias similar to those evoked by exposure to thin ideals in Western culture and that this bias may contribute to the development of body image disorders. It is true that for visual adaptation to constitute an experimental model of body image distortion, some overlap of the representation of self and others is required to allow the transfer of aftereffects from media images to the perception of one’s own body. However, if visual adaptation to bodies truly modulates one’s own body image, then it should also differ from the general effect adaptation has on all images of bodies. If both the item being tested (own body) and the probe (other bodies) are equally affected by adaptation, the relative difference between them should not change. If all bodies are equally affected, adaptation to extreme body types cannot serve as a sufficient explanation for own body image distortion. If, however, adaptation affects perception of one’s own body and other bodies differently, it may suggest that it affects higher level representation of one’s own body and not only the experience of the visual image. Here, we found equally strong aftereffects for judgments about one’s own body and other people’s bodies. Thus, our results provide no evidence that body size adaptation has an effect that shows specificity to one’s body image.

Brooks et al. (2016) showed stronger body size aftereffects when the identity between the adaptor and test stimuli matched, regardless of whether both corresponded to images of the participant’s own body or the body of an unknown other. Nonetheless, they also found some transfer in body size aftereffects between identities, so that adaptation to a fat or thin body of an unknown other affected the perception of images of their own body at test, and vice versa. These effects suggest a partial dissociation of the neural mechanisms encoding body size for self and other. However, as noted by the authors themselves, the use of only two identities (“own body” and “the body of an unfamiliar other”) does not allow to rule out the possibility that the observed effects are not specific to the self, but rather would have been observed regardless of the two specific identities used. In the present study, we address this issue by requesting judgments about the same test stimuli throughout, but using as criteria different internal representations (i.e., an image of the body self or the body of another person), varying, therefore, the context. Interestingly, Winkler and Rhodes (2005) found that attractiveness aftereffects were observed following adaptation to extremely thin bodies but not following adaptation to fat bodies, whereas no such asymmetry was found when participates were asked to judge the perceived normality of the test image. This asymmetry suggests that strength of adaptation aftereffects in some cases may be mediated by the context of the task. Furthermore, de la Rosa et al. (2014) found modulation of action adaptation aftereffects across two conditions that had identical adapting and test stimuli but differed with respect to the information that was provided prior to the adaptation, i.e., the social context. However, in our study, the context of the specific judgment being made (i.e., about one’s own body or about someone else’s) did not affect the magnitude of the adaptation aftereffects.

There are some clear limitations of the present study. Our study reports null findings, which have an ambiguous status in the field, not allowing to draw very strong conclusions. However, we found consistent results across experiments and in all three experiments, we report Bayes factors that provided moderate support in favor of the null hypothesis. Furthermore, the use of only a single identity stimuli meant that adaptation in our study was specific to this identity (see Brooks et al., 2016). Future studies should further investigate this topic using multiple identities that share only the property of adiposity.

Our study investigated short-term effects of relatively short adaptation period (a couple of minutes). It is possible that longer term adaptation affects body representations in a way that goes beyond pure visual perception. One of the main characteristics of both low-level and high-level perceptual aftereffects is that their magnitude depends on the length of adaptation (Leopold et al., 2005). Recent research on lower level visual adaptation aftereffects has suggested that there may be qualitatively distinct mechanisms that underlie adaptation over very short-term and longer term time scales (e.g., Vul et al., 2008; Bao and Engel, 2012; Bao et al., 2013). It is therefore possible that long-term exposure to extreme body types could produce a different pattern of results to those we found in this study. Future studies should examine whether prolonged visual adaptation can cause aftereffects in another modality, related to body representation, e.g., touch.

Our results also do not allow us to distinguish whether the aftereffects we report reflect changes in the actual perception of body stimuli or higher level decisional processes used to make judgments about these stimuli. Recent work has suggested that many effects previously interpreted as perceptual may actually reflect decisional processes (e.g., Morgan et al., 2011; Firestone and Scholl, 2013). The psychophysical methods used in this study, like nearly all other studies of “high-level” adaptation aftereffects, do not allow these interpretations to be distinguished (cf. Storrs, 2015).

## Data Availability Statement

The datasets generated for this study are available on request to the corresponding author.

## Ethics Statement

This study was carried out in accordance with the recommendations of the ethics board of the Department of Psychological Sciences, Birkbeck, University of London, with written informed consent from all subjects. All subjects gave written informed consent in accordance with the Declaration of Helsinki. The protocol was approved by the ethics board of the Department of Psychological Sciences, Birkbeck, University of London.

## Author Contributions

KA, EA, and ML designed the study and wrote the manuscript. KA collected the data. KA analyzed the data under supervision of EA and ML.

### Conflict of Interest

The authors declare that the research was conducted in the absence of any commercial or financial relationships that could be construed as a potential conflict of interest.

## Footnotes

^1^Because of a programming error, in some subjects, some of the PSEs that came close to the upper limit of possible stimuli could have suffered from a ceiling effect. In all cases, only one PSE per condition was affected (two were obtained), which made it possible to remove the affected PSEs in an additional analysis. Removing the affected PSEs (10.6% of all PSEs) did not change the overall pattern of the results. Imputing the affected PSEs using regression analysis also yielded very similar results.
